# Investigating the relationship between inhibitory control and dietary adherence among patients with type 2 diabetes mellitus based on subjective and objective measures

**DOI:** 10.1038/s41387-023-00252-4

**Published:** 2023-11-22

**Authors:** Na Liu, Chunni Heng, Yi Cui, Di Wu, Ling Li, Mengge Bai, Yanxue Guo, Wen Wang, Yinling Zhang

**Affiliations:** 1 Department of Nursing, Air Force Medical University, Xi’an, China; 2 Department of Endocrinology, the Second Affiliated Hospital of Air Force Medical University, Xi’an, China; 3 Department of Military Medical Psychology, Air Force Medical University, Xi’an, China; 4 Department of Endocrinology, the Second Medical Center of Chinese PLA General Hospital, Beijing, China; 5 Department of Radiology, Functional and Molecular Imaging Key Lab of Shaanxi Province, Tangdu Hospital, Fourth Military Medical University, Xi’an, China

**Keywords:** Type 2 diabetes, Nutrition therapy, Risk factors, Cognitive control

## Abstract

**Background:**

Dietary management has been recommended as the cornerstone of type 2 diabetes mellitus (T2DM) management. However, low adherence to dietary recommendations has been identified in both developed and developing countries. Previous research suggests that inhibitory control influences eating behavior, but few studies have been conducted in patients with T2DM. Thus, we aimed to explore the relationship between inhibitory control and dietary adherence among patients with T2DM.

**Methods:**

A total of 393 patients with T2DM from the endocrinology departments of three tertiary hospitals in China were enrolled by the convenience sampling method. Dietary adherence was measured by the Dietary Behavior Adherence Scale for Patients with Type 2 Diabetes Mellitus. Additionally, inhibitory control was subjectively measured by the Behavior Rating Inventory of Executive Function-Adult version (BRIEF-A) and objectively assessed by the stop signal task (SST) and the Stroop task. The relationship between inhibitory control and dietary adherence was analyzed using Pearson correlation analysis and hierarchical regression analysis.

**Results:**

Subjectively measured inhibitory control had a significant predictive effect for dietary adherence after controlling for demographic and clinical variables. Adding the inhibitory control variable to the regression equation resulted in the following values: overall model *F* (19, 373) = 7.096, *p* < 0.001, increase in *R*^2^ value by 0.069, change in *F* (1, 373) = 35.219, *p* < 0.001. Similarly, the performance of the Stroop task had a significant predictive effect for dietary adherence to some foods, i.e., carbohydrate and fat. Adding the Stroop effect variable to the regression equation resulted in the following values: overall model *F* (19, 81) = 2.848, *p* = 0.005, increase in *R*^2^ value by 0.060, change in *F* (1, 81) = 8.137, *p* = 0.006.

**Conclusions:**

Inhibitory control was a predictor of dietary adherence in patients with T2DM. Future interventions should investigate whether inhibitory control training results in the improvement of dietary adherence in patients with T2DM.

## Introduction

Diabetes mellitus (DM) has been one of the most predominant chronic diseases in the current century [1]. In 2021, approximately 537 million (10.5%) adults aged 20–79 years were living with diabetes worldwide, and this number is predicted to rise to 783 million (12.2%) by 2045 [2]. With the aging of the population and the increasing prevalence of obesity, diabetes has become a major public health problem in China with 140.9 million patients with diabetes, making it the country with the highest number of diabetes patients [2]. T2DM is the most common type, accounting for more than 90% of all diabetes cases [3]. Dietary management is the keystone of T2DM management and is usually recommended as the first step of diabetes management; it plays a pivotal role in maintaining glycemic control and reducing the risk of diabetes-related complications [4, 5]. Guidelines for diabetes management across different countries have emphasized the significance of dietary management in patients with T2DM [1, 6, 7]. However, adherence to dietary recommendations continues to be a highly challenging task for patients with T2DM, as it usually requires long-term resistance to the patient’s food cravings and preferences and the temptation of delicious food [1, 8, 9], which relies on the patient’s inhibitory control [10]. Therefore, a deeper understanding of the relationship between inhibitory control and dietary adherence is essential to improve the dietary management of T2DM patients.

Inhibitory control, the core component of executive function, is an important basis for high-order cognitive functions, such as resisting temptation and making long-term decisions [11]. It refers to the ability to control one’s attention, behavior, thoughts, and/or emotions; override a strong internal predisposition or external lure; and instead do what is more appropriate or needed [12]. It is generally acknowledged that inhibitory control includes response inhibition and interference inhibition [12]; the former mainly refers to suppressing the dominant response, often measured by the stop signal task (SST), whereas the latter focuses on suppressing irrelevant information, as estimated by the Stroop task [13]. Additionally, inhibitory control can also be subjectively measured by the Behavior Rating Inventory of Executive Function-Adult version (BRIEF-A) [14]. Although task-based measures have generally been considered the gold standard for measuring inhibitory control, they lack ecological validity [15]. A subjective questionnaire used to measure an individual’s perception of inhibitory control in their daily life has more ecological validity, despite the lack of objectivity. Importantly, both task-based and questionnaire measures may provide insight into different aspects of inhibitory control [15]. Therefore, combining subjective and objective measures to assess inhibitory control can not only compensate for the shortcomings of each measure itself but also provide complementary information about inhibitory control. To the best of our knowledge, no studies have presented this type of combined data among T2DM patients thus far.

Inhibitory control is considered a key factor in successful food intake control [16]. Numerous studies have shown that impaired inhibitory control negatively affects eating behavior [10, 11, 16]. Individuals with poorer inhibitory control tend to have more difficulty resisting food temptations and are more likely to choose high-calorie foods, which results in behaviors or outcomes associated with poor self-management behaviors (e.g., overeating, weight gain, and obesity) [11, 17–20]. In contrast, inhibitory control positively predicted the intake of fruits and vegetables in overweight and obese patients [21]. Intervention studies have also shown that training in the inhibitory control of food cues can reduce individuals’ preference for high-calorie food and reduce the amount of snacks consumed in sham taste tests [22, 23]. However, previous studies mainly focused on restrictive dieters [24], eating disorders [25], or overweight and obese patients [26] and rarely involved people with chronic diseases that require long-term strict dietary management, such as diabetes. According to the temporal self-regulation theory, inhibitory control, as a proximal factor of health-promoting behaviors (e.g., diet management, exercise, weight control, and regular check-ups), can have direct or indirect effects on health behaviors of people with chronic diseases [27]. Therefore, we speculate that inhibitory control could be a potentially influencing factor of dietary adherence in patients with T2DM. The present study aimed to verify the relationship between inhibitory control and dietary adherence among patients with T2DM.

Our study employed subjective questionnaire and objective task-based measures to assess inhibitory control and to comprehensively investigate the relationship between inhibitory control and dietary adherence in T2DM patients. First, we used BRIEF-A to examine whether patients’ perception of inhibitory control in their daily life was related to dietary adherence. Next, we utilized SST and the Stroop task to examine whether patients’ objective response inhibition ability and interference inhibition ability, respectively, were related to dietary adherence.

## Materials and methods

### Participants and procedures

The convenience sampling method was used to recruit patients with T2DM from the endocrinology departments of three tertiary hospitals in China from March to September 2022 for this cross-sectional descriptive study. The inclusion criteria were as follows: T2DM patients who (1) met the 1999 WHO diagnostic criteria for diabetes; (2) had a course of disease ≥6 months; (3) had an age ≥18 years old; (4) had good verbal communication and understanding skills; (5) had normal vision or corrected vision, no color blindness or color asthenia; (6) had normal finger function and ability to do key reactions; (7) had a MoCA score ≥25; and (8) had given informed consent and were willing to participate in the study. Patients who (1) had a history of cerebrovascular disease or other central nervous system injury and (2) had difficulty completing the questionnaire or the computer-based cognitive measurement tasks were excluded. As effect size was 0.3, test level α was 0.05, and power was 0.80, the minimal number of patients was 82 patients.

Data collection consisted of two phases. In the first phase, the questionnaire data were collected. First, the researchers explained the purpose and significance of the study to participants using uniform instructions in a face-to-face manner and obtained their written informed consent. Then, the cognitive screening of the participants was completed in a quiet and comfortable environment. It took 8–10 min for the participants to complete the MoCA scale in question–answer form. During this process, the researchers scored the participants’ answers according to the instructions. Finally, it took approximately 10–15 min to complete the remaining questionnaires, including a sociodemographic questionnaire, BRIEF-A, and the Dietary Behavior Adherence Scale for Patients with Type 2 Diabetes Mellitus. In order to minimize possible common method biases, questionnaire items were randomly arranged in this study. After receiving the questionnaires, the researchers scrutinized all items of each questionnaire to ensure that there were no omissions or errors. If there was any omission or error, the participants were asked to complete or modify it.

In the second phase, inhibitory control was objectively measured. A total of 108 of the participants who completed the questionnaire were willing to continue to participate in the next two objective inhibitory control tasks, i.e., SST and Stroop task. First, the researchers introduced the rules and precautions of the experiment to the patients until they understood and then guided the patients into the experimental practice part to familiarize themselves with the specific operation process for the entire experiment. Formal trials were independently performed by the patients without any investigator guidance. This study was approved by the Ethics Committee of Air Force Medical University (No. 202206-02).

### Measures

#### Demographic and clinical variables

##### Sociodemographic questionnaire

The questionnaire was self-designed by the investigators for the purpose of the study. The content of the questionnaire included age, gender, education level, monthly family income, course of diabetes, history of hypoglycemia in the past year, diabetic complications, treatment method, and BMI.

#### Cognitive screening

##### Montreal Cognitive Assessment (MoCA)

The MoCA is an internationally recognized standard examination tool for screening cognitive impairment. “Expert Consensus on Cognitive Impairment in Diabetic Patients” recommends the MoCA as a screening tool for mild cognitive impairment in diabetic patients [28]. The scale contains 11 items in 8 cognitive domains, such as visuospatial structure, executive ability, attention, memory, language function, abstract thinking, calculation and orientation, and can quickly screen mild cognitive function. The total score is 30 points, and higher scores indicate better cognitive function. A MoCA score ≥25 indicates a normal cognitive level [29].

#### Dietary adherence

##### The Dietary Behavior Adherence Scale for Patients with Type 2 Diabetes Mellitus

The questionnaire was developed by a Chinese scholar, Zhao Qiuli, and colleagues [30], through literature review and interviews in combination with the “Dietary Guidelines for Chinese Residents” and “Guidelines for the Prevention and Treatment of Type 2 Diabetes in China.” It is mainly used to measure the dietary behavior adherence of T2DM patients. Considering the differences in diet culture within and outside of China, this scale was suitable for this study. The scale includes the following 5 dimensions, comprising 23 items: diet self-regulation (5 items), carbohydrate and fat adherence behavior (5 items), oil and salt adherence behavior (4 items), fruit and vegetable adherence behavior (5 items) and cooking and eating habits (4 items). A 5-point Likert scoring method was used for each item: never = 1, rarely = 2, sometimes = 3, often = 4, and always = 5. The total score of the scale and the scores of each dimension were calculated; higher scores indicated better dietary behavior adherence. In this study, the overall Cronbach’s *α* coefficient of the scale was 0.891 (95% CI, 0.875–0.906), and the Cronbach’s *α* coefficients of the five dimensions ranged from 0.729 (95% CI, 0.684–0.769) to 0.830 (95% CI, 0.801–0.855).

### Inhibitory control

#### Subjective measure

##### The Behavior Rating Inventory of Executive Function-Adult Version (BRIEF-A)

The inhibitory control subscale in BRIEF-A was used to measure the level of inhibitory control. The BRIEF-A is a standardized self-report questionnaire that measures individuals’ everyday executive function by assessing the behaviors associated with executive function deficits in everyday life in adults [31]. The scale included the following nine subscales: inhibitory control (8 items), shift (6 items), emotional control (10 items), self-monitoring (6 items), initiative (8 items), working memory (8 items), plan/organize (10 items), task monitor (6 items) and organization of materials (8 items). The BRIEF-A contains 75 items, of which five infrequency items are designated to detect atypical responses, and 70 items assess executive function. All items were rated on a three-point scale from 1 to 3. The total points of each subscale and index were transformed into age-appropriate standard scores (*T*-scores) based on normative data [31]. Higher *T*-scores reflect greater impairments in executive function. It has been proven that the BRIEF-A has good validity and reliability in China [32]. According to the purpose of the current study, the inhibitory control subscale was statistically analyzed, and the Cronbach’s *α* coefficient was 0.797 (95% CI, 0.765–0.826).

#### Objective measures

##### Stop signal task (SST)

**Fig. 1:**
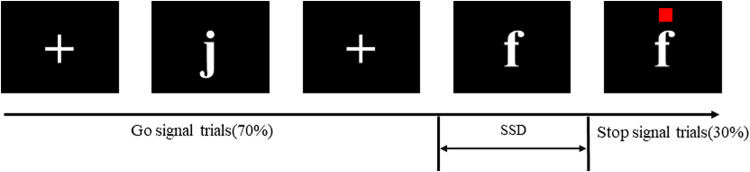
Schematic representation of the stop signal task.

The SST is a classical experimental paradigm to evaluate response inhibition ability [33]. The basic experimental process consists of two types of tasks: the go task and the stop task. The go task required the subjects to quickly make a selective key response after seeing the go signal (such as pressing the “F” key with the left index finger when “f” appeared and pressing the “J” key with the right index finger when “j” appeared). During the stop task, an obvious red dot appeared at a certain time interval (stop signal delay, SSD) after the stop signal appeared, at which time the subject was required to inhibit the impulse to press the key and stop the button reaction. The stop task appeared randomly throughout the experiment and usually accounted for 20–30% of the total task [34]. In this study, the proportion of stop signal trials in the task was set to 30% to obtain more inhibition trials [35]. The task consisted of a total of 200 trials, including 140 go signal trials and 60 stop signal trials. Stimuli were presented in the center of the screen, with a fixation point (+) appearing 250 ms before each stimulus and a 1000 ms time interval after each response keystroke. In the go task, the maximum presentation time of the go signal was 1250 ms, and if the participant did not press the button in time, the screen reminded them that their response was “too slow.” In the stop task, the stop signal appeared slightly later than the go signal, and if the inhibition was successful (no key pressed), the stimulus presentation time was also a maximum of 1250 ms. The initial value of the SSD in this task was set to 250 ms, and the SSD was automatically adjusted by the tracking method. If key impulse inhibition was successful, the SSD in the next stop task was increased by 50 ms. If key impulse suppression failed, the SSD in the next stop task was reduced by 50 ms to ensure that the successful inhibition rate of the participant was approximately equal to 50% (Fig. 1).

The go response time (GoRT) of the go task and SSD were recorded, and the stop signal response time (SSRT) was calculated according to the independent race model as an evaluation index of response inhibition ability [36]. The specific calculation method is as follows: SSRT = average GoRT − average SSD. A smaller SSRT indicates a greater response inhibition ability.

#### Stroop task

**Fig. 2:**
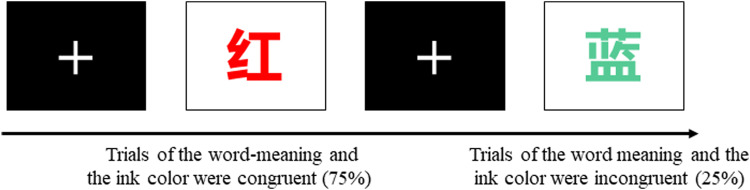
Schematic representation of the Stroop task.

The Stroop task is a classic paradigm for assessing individual interference inhibition ability [37]. The color-word Stroop task was used in this study. The experimental materials were composed of four color words, including “red,” “blue,” “green,” and “yellow.” There were two conditions: the congruent condition and the incongruent condition. The congruent condition indicates that the word meaning and the ink color were congruent, including “red” in red ink, “green” in green ink, “yellow” in yellow ink, and “blue” in blue ink. The incongruent condition indicates that the word meaning and the ink color were incongruent, such as “blue” in green ink. There were a total of 256 trials, which were randomly presented in the center of the screen. The ratio of consistent trials to inconsistent trials was 3:1. The subjects were asked to press the corresponding key according to the actual color of the word (press the “F” key for red, the “G” key for blue, the “J” key for green, and the “K” key for yellow). The corresponding key fingers were the left middle finger, left index finger, right index finger and right middle finger, respectively (Fig. 2).

The reaction times of congruent and incongruent trials were recorded, and the Stroop effect or conflict effect (the difference in reaction time between congruent trials and incongruent trials) was calculated to evaluate the interference inhibition ability. A smaller Stroop effect indicates greater interference inhibition ability.

### Statistical analyses

The collected data were entered by Epidata Version 3.0 software and analyzed by SPSS Version 23.0. We performed descriptive statistical analyses for sociodemographic characteristics, dietary adherence, and inhibitory control based on subjective and objective measures. With the data meet the normal distribution, an independent *t* test or one-way ANOVA was performed to compare dietary adherence among different sociodemographic subgroups. Pearson correlation analysis and hierarchical regression analysis were performed to analyze the relationship between inhibitory control and dietary adherence. A *p* level of <0.05 was considered statistically significant.

## Results

### Study participants

**Fig. 3:**
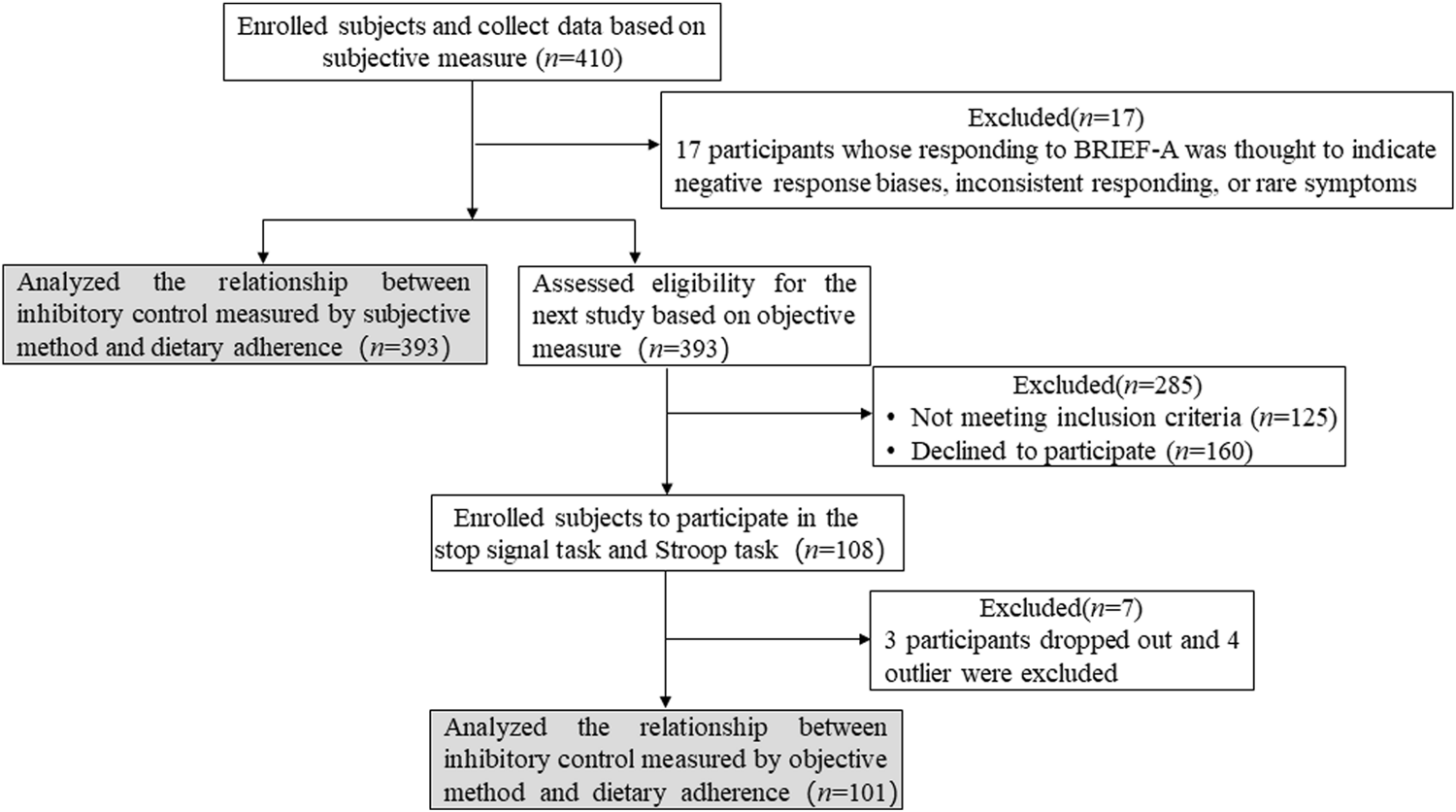
CONSORT flowchart of the study.

A total of 410 participants were enrolled from the endocrinology departments of three tertiary hospitals in China from March to September 2022. However, 17 participants whose responses to BRIEF-A were thought to indicate negative response biases, inconsistent responding, or rare symptoms were excluded from this analysis. Among the 393 participants, 108 participants continued to participate in the stop signal task and the Stroop task, but 3 participants dropped out and 4 outlier participants were excluded (Fig. 3).

### Sociodemographic characteristics and comparisons of dietary adherence of the participants

Table 1 presents the sociodemographic characteristics and the results of the comparisons of dietary adherence of the participants. A total of 393 T2DM patients were enrolled in this study, including 310 males (78.88%) and 83 females (21.12%). The mean age of the participants was 51.55 ± 11.45 years (range from 20 to 81 years). The mean course of diabetes among the participants was 9.46 ± 6.26 years (range from 6 months to 30 years). A total of 41.98% of the patients reported one or more episodes of hypoglycemia in the past year. More than half of the patients were overweight or obese.

**Table 1:** Sociodemographic characteristics and comparisons of dietary adherence of the participants (*n* = 393).

Variables	*n* (%)	*M* ± SD^a^	*t*/*F*	*p*
Age			6.935^b^	0.001
<45	105 (26.72)	3.14 ± 0.64		
45–60	182 (46.31)	3.19 ± 0.60		
≥60	106 (26.97)	3.42 ± 0.54		
Gender			−2.591^c^	0.01
Male	310 (78.88)	3.20 ± 0.60		
Female	83 (21.12)	3.39 ± 0.59		
Education level			11.047^b^	<0.001
Junior high school and below	79 (20.10)	2.94 ± 0.57		
High school	120 (30.53)	3.19 ± 0.65		
Junior college	83 (21.12)	3.39 ± 0.54		
Bachelor or above	111 (28.24)	3.38 ± 0.52		
Monthly family income (Yuan)			1.689^b^	0.186
<3000	101 (25.70)	3.20 ± 0.62		
3000–5000	115 (29.26)	3.17 ± 0.63		
≥5000	177 (45.04)	3.30 ± 0.56		
Course of diabetes			1.689^b^	0.186
≤4 years	130 (33.08)	3.21 ± 0.63		
5–10 years	129 (32.82)	3.18 ± 0.58		
>10 years	134 (34.10)	3.31 ± 0.59		
Diabetic complication			0.966^c^	0.335
Yes	72 (18.32)	3.30 ± 0.61		
No	321 (81.68)	3.22 ± 0.60		
Hypoglycemia in the past year			2.856^c^	0.005
Yes	165 (41.98)	3.34 ± 0.58		
No	228 (58.02)	3.16 ± 0.60		
Treatment method			0.461^b^	0.709
Oral hypoglycemic agents	146 (37.15)	3.25 ± 0.61		
Insulin	150 (38.17)	3.20 ± 0.58		
Insulin and oral hypoglycemic agents	56 (14.25)	3.29 ± 0.56		
Diet and exercise	41 (10.43)	3.27 ± 0.71		
BMI (kg/m^2^)			4.713^b^	0.003
≤18.4	12 (3.05)	3.30 ± 0.44		
18.5–23.9	158 (40.20)	3.36 ± 0.57		
24–27.9	157 (39.95)	3.17 ± 0.62		
≥28	66 (16.79)	3.08 ± 0.59		

Statistically significant between groups at *p* < 0.05.

*BMI* body mass index.

^a^Scores of dietary adherence.

^b^One-way ANOVA.

^c^Two independent samples *t* test.

For dietary adherence among the patients with T2DM, homogeneity of variance was observed among the groups. Statistically significant differences were observed in age (*F* (2, 390) = 6.94, *p* = 0.001, *η*^2^ = 0.04), gender (*t* = −2.59, *p* = 0.010, *d* = 0.02), education level (*F* (3, 389) = 11.05, *p* < 0.001, *η*^2^ = 0.08), hypoglycemia in the past year (*t* = 2.86, *p* = 0.005, *d* = 0.02), and BMI (*F* (3, 389) = 4.71, *p* = 0.003, *η*^2^ = 0.04).

### Correlation analysis of inhibitory control and dietary adherence

Table 2 presents the correlation analysis of inhibitory control measured by subjective methods and dietary adherence among the participants. The results showed that inhibitory control was negatively correlated with total score and all dimensions of dietary adherence (*p* < 0.05).

**Table 2:** Pearson correlation analysis of inhibitory control measured by subjective method and dietary adherence (*n* = 393).

	1	2	3	4	5	6	7
1. Total score	1						
2. Fruit and vegetable	0.761**	1					
3. Oil and salt	0.773**	0.486**	1				
4. Carbohydrate and fat	0.724**	0.378**	0.526**	1			
5. Diet self-regulation	0.831**	0.535**	0.534**	0.484**	1		
6. Cooking/eating habits	0.716**	0.528**	0.427**	0.335**	0.525**	1	
7. Inhibitory control	−0.312**	−0.250**	−0.196**	−0.227**	−0.221*	−0.312**	1
*M*	3.24	3.25	3.01	3.04	3.35	3.55	51.33
SD	0.6	0.7	0.84	0.75	0.88	0.76	9.69

Total score = Total score of dietary adherence; Fruit and vegetable = Fruit and vegetable adherence behavior; Oil and salt = Oil and salt adherence behavior; Carbohydrate and fat = Carbohydrate and fat adherence behavior.

*M* mean, *SD* standard deviation.

*p < 0.05; **p < 0.01.

Table 3 presents the correlation analysis of inhibitory control measured by objective methods and dietary adherence among the participants. The results showed that the Stroop effect was significantly negatively related to carbohydrate and fat adherence behavior (*r* = −0.244, *p* = 0.014), which indicated that the stronger the interference inhibition ability was, the better the dietary adherence to carbohydrate and fat foods. However, performance in the SST did not have a statistically significant association with total score or any dimension of dietary adherence (*p* > 0.05).

**Table 3:** Pearson correlation analysis of inhibitory control measured by objective method and dietary adherence (*n* = 101).

	SSRT	Stroop effect
Total score	−0.098	−0.098
Fruit and vegetable	−0.073	−0.023
Oil and salt	0.042	−0.077
Carbohydrate and fat	−0.148	−0.244*
Diet self-regulation	−0.079	−0.04
Cooking/eating habits	−0.095	0.042
*M*	300.65	102.12
SD	49.88	62.21

Total score = Total score of dietary adherence; Fruit and vegetable = Fruit and vegetable adherence behavior; Oil and salt = Oil and salt adherence behavior; Carbohydrate and fat = Carbohydrate and fat adherence behavior.

*M* mean, *SD* standard deviation, *SSRT* stop signal response time.

*p < 0.05.

### Hierarchical regression analysis of dietary adherence

Table 4 presents the hierarchical regression analysis of dietary adherence among the participants. A hierarchical regression analysis was conducted with demographic and clinical variables as the control variables, inhibitory control as the independent variable, and dietary adherence as the dependent variable. The results showed that inhibitory control had a significant predictive effect for dietary adherence after controlling for the demographic and clinical variables (*p* < 0.001). Adding the inhibitory control variable in model 2 resulted in the following values: overall model *F* (19, 373) = 7.096, *p* < 0.001, increase in *R*^2^ value by 0.069, change in *F* (1, 373) = 35.219, *p* < 0.001. These results were statistically significant.

**Table 4:** Hierarchical regression analysis of dietary adherence (*n* = 393).

	Model 1					Model 2				
	*B*	Std. error	*β*	95% CI	*p*	*B*	Std. error	*β*	95% CI	*p*
**Constant**	3.924	0.147	–	(3.635, 4.212)	<0.001	4.700	0.192	–	(4.323, 5.077)	<0.001
Age (take ≥60 as reference)										
<45	−0.264	0.091	−0.195	(−0.444, −0.085)	0.004	−0.233	0.087	−0.172	(−0.405, −0.061)	0.008
45–60	−0.210	0.071	−0.174	(−0.349, −0.07)	0.003	−0.196	0.068	−0.163	(−0.330, −0.063)	0.004
Gender (take female as reference)										
Male	−0.260	0.073	−0.177	(−0.402, −0.117)	<0.001	−0.231	0.070	−0.157	(−0.368, −0.094)	0.001
Education level (take bachelor or above as reference)										
Junior high school and below	−0.456	0.088	−0.305	(−0.629, −0.283)	<0.001	−0.373	0.085	−0.249	(−0.541, −0.206)	<0.001
High school	−0.275	0.080	−0.211	(−0.432, −0.118)	0.001	−0.204	0.077	−0.156	(−0.356, −0.052)	0.009
Junior college	−0.007	0.082	−0.005	(−0.169, 0.155)	0.934	0.012	0.079	0.008	(−0.143, 0.167)	0.880
Diabetic complication (take no as reference)										
Yes	0.194	0.076	0.125	(0.044, 0.344)	0.012	0.195	0.073	0.126	(0.051, 0.339)	0.008
BMI (take 18.5–23.9 as reference)										
≤18.4	−0.165	0.170	−0.047	(−0.499, 0.169)	0.332	−0.227	0.163	−0.065	(−0.547, 0.093)	0.165
24–27.9	−0.157	0.065	−0.128	(−0.284, −0.030)	0.016	−0.167	0.062	−0.137	(−0.289, −0.045)	0.007
≥28	−0.273	0.087	−0.170	(−0.444, −0.103)	0.002	−0.264	0.083	−0.165	(−0.428, −0.010)	0.002
Inhibitory control						−0.016	0.003	−0.274	(−0.022, −0.011)	<0.001
*R*^2^	0.196					0.265				
Adj *R*^2^	0.157					0.228				
*F*	*F* (18, 374) = 5.070**					*F* (19, 373) = 7.096**				
^Δ^*R*^2^	0.196					0.069				
^Δ^*F*	^Δ^*F* (18, 374) = 5.070**					^Δ^*F* (1, 373) = 35.219**				

*B* = unstandardized regression coefficient; Std. error = standard error; *β* = standardized regression coefficient; *R*^2^ = coefficient of determination; Adj *R*^2^ = Adjusted coefficient of determination; ^Δ^*R*^2^ = change of coefficient of determination; ^Δ^*F* = *F* change.

*BMI* body mass index.

**p < 0.01.

Table 5 presents the hierarchical regression analysis of carbohydrate and fat adherence behavior among the participants. A hierarchical regression analysis was conducted with demographic and clinical variables as the control variables, Stroop effect as the independent variable, and carbohydrate and fat adherence behavior as the dependent variable. The results showed that the Stroop effect had a significant predictive effect for carbohydrate and fat adherence behavior after controlling for the demographic and clinical variables (*p* = 0.006). Adding the Stroop effect variable in model 2 resulted in the following values: overall model *F* (19, 81) = 2.848, *p* = 0.005, increase in *R*^2^ value by 0.060, change in *F* (1, 81) = 8.137, *p* = 0.006. These results were statistically significant.

**Table 5:** Hierarchical regression analysis of carbohydrate and fat adherence behavior (*n* = 101).

Variables	Model 1					Model 2				
	*B*	Std. error	*β*	95% CI	*p*	*B*	Std. error	*β*	95% CI	*p*
Constant	4.006	0.355	–	(3.299, 4.712)	<0.001	4.270	0.353	–	(3.567, 4.972)	<0.001
Education level (take bachelor or above as reference)										
Junior high school and below	−0.453	0.217	−0.260	(−0.884, −0.022)	0.040	−0.502	0.208	−0.288	(−0.917, −0.087)	0.018
High school	−0.363	0.205	−0.216	(−0.77, 0.044)	0.080	−0.387	0.197	−0.230	(−0.778, 0.004)	0.053
Junior college	−0.084	0.183	−0.052	(−0.449, 0.281)	0.647	−0.113	0.176	−0.070	(−0.464, 0.237)	0.523
Treatment method (take diet and exercise as reference)										
Oral hypoglycemic agents	−0.325	0.242	−0.234	(−0.808, 0.157)	0.183	−0.336	0.233	−0.242	(−0.799, 0.126)	0.152
Insulin	−0.034	0.270	−0.021	(−0.57, 0.502)	0.900	−0.037	0.259	−0.022	(−0.552, 0.477)	0.886
Insulin and oral hypoglycemic agents	−0.694	0.271	−0.374	(−1.233, −0.155)	0.012	−0.698	0.260	−0.376	(−1.215, −0.180)	0.009
Stroop effect						−0.003	0.001	−0.254	(−0.005, −0.001)	0.006
*R*^2^	0.340					0.401				
Adj *R*^2^	0.195					0.260				
*F*	*F* (18, 82) = 2.350**					*F* (19, 81) = 2.848**				
^Δ^*R*^2^	0.340					0.060				
^Δ^*F*	^Δ^*F* (18, 82) = 2.350**					^Δ^*F* (1, 81) = 8.137**				

*B* = Unstandardized regression coefficient; Std. error = standard error; *β* = standardized regression coefficient; *R*^2^ = coefficient of determination; Adj *R*^2^ = Adjusted coefficient of determination; ^Δ^*R*^2^ = change of coefficient of determination; ^Δ^*F* = *F* change.

**p < 0.01.

## Discussion

The current study validated the relationship between dietary adherence and inhibitory control measured by subjective and objective methods among patients with type 2 diabetes mellitus. These findings provide a scientific theoretical basis and a new intervention target to improve dietary adherence among patients with T2DM. To the best of our knowledge, the current study was one of the first to comprehensively analyze the relationship between inhibitory control and dietary adherence in patients with T2DM by using both subjective and objective measures in the same study.

Dietary adherence refers to the consistency between patients’ dietary behavior and their doctors’ advice in the process of receiving treatment [38]. Dietary adherence is the most important index to measure the dietary management behavior of patients with diabetes [30]. By evaluating the dietary adherence of patients with T2DM, targeted dietary guidance can be given to patients, which is conducive to delaying the occurrence of diabetic complications and improving the quality of life of patients [30]. However, dietary adherence in patients with T2DM is low, and it is influenced by many factors. This study showed that dietary adherence was significantly different among different categories of age, gender, education level, hypoglycemia in the past year, and BMI. Further analysis showed that female elderly patients with a higher education level and a history of hypoglycemia in the past year tended to have higher dietary adherence. Patients with a BMI in the overweight range (a BMI of 24 or more) were more likely to have low dietary adherence.

In addition, this study also found a significant correlation between inhibitory control and dietary adherence among patients with T2DM based on both subjective and objective measures. The results showed that inhibitory control measured by subjective methods was positively correlated with dietary adherence. Similarly, the performance of the Stroop task was significantly positively related to dietary adherence to some foods, i.e., carbohydrate and fat. Additionally, inhibitory control had a significant predictive effect for dietary adherence when controlling for demographic and clinical variables. To the best of our knowledge, this is the first study to comprehensively explore the relationship between inhibitory control and dietary adherence among patients with T2DM by combining both subjective and objective measures. Although recent neuroimaging findings suggest that the two sets of measures share a common neuroanatomical substrate [39], some suggest that performance-based tasks assess underlying skills, while rating scales assess the application of those skills in daily life [40]. Thus, they measure different aspects of inhibitory control. In addition, inhibitory control is a multifaceted concept, and different types of inhibitory control can be reflected by different objective cognitive tests; for example, the stop signal task assesses a person’s response inhibition ability, and the Stroop task assesses a person’s interference inhibition ability. Therefore, although both subjective and objective measures of inhibitory control were significantly associated with dietary adherence, the results were not completely consistent.

In the case of specific results relating to subjective measures, the T score of inhibitory control was negatively correlated with the total score and all dimensions of dietary adherence, and inhibitory control had a significant predictive effect for dietary adherence when controlling for demographic and clinical variables. Namely, the poorer the level of inhibitory control, the worse the dietary adherence in patients with T2DM. This study is one of the first to reveal the relationship between inhibitory control and dietary adherence in patients with T2DM by using rating scales. In other words, the patients’ perceived (i.e., subjective) inhibitory control dysfunction can be utilized by medical staff in determining their patients’ specific challenges in following diabetic diet control. This result has important clinical significance for improving dietary adherence among patients with T2DM.

In the case of specific results related to objective measures, this study revealed a significant correlation between inhibitory control measured by the Stroop task and dietary adherence in patients with T2DM. Specifically, the Stroop effect was significantly negatively related to carbohydrate and fat adherence behavior, and the Stroop effect had a significant predictive effect when controlling for demographic and clinical variables, which indicated that the stronger the interference inhibition ability was, the better the dietary adherence to carbohydrate and fat foods. To the best of our knowledge, this is the first study to reveal the relationship between interference inhibition and dietary adherence among patients with T2DM. For patients with diabetes, a low-carbohydrate, low-fat, light diet contributes to controlling blood glucose, but these foods are often not delicious enough to bring pleasure to patients. Thus, when faced with a variety of food temptations, some patients with poor interference inhibition ability may show self-management relaxation and compromise to enjoy the momentary pleasure brought by food, thus demonstrating decreased dietary adherence. Therefore, when treating patients with poor dietary adherence, health care providers should consider the underlying factor of impaired inhibitory control.

However, performance of the SST did not have a statistically significant association with dietary adherence, which was inconsistent with a previous study [41]. Zhu and her colleagues found that there were significant differences in response inhibition among T2DM patients with different dietary adherence [41]. The reason for the inconsistent results may be because the experimental stimuli were different. In our study, the stimuli were “j” and “f,” which mainly measured the general response inhibition, whereas in Zhu’s study, the stimuli were pictures of food, which mainly measured the food-specific response inhibition. Many research findings indicate that dietary behavior regulation is related to poor response inhibition that is food-specific, rather than general response inhibition [42–44].

However, there were several limitations in our study that should be considered. First, the generalizability of our findings is limited since all of the patients were recruited from only three hospitals. Second, we could not make inferences about the causality between inhibitory control and dietary adherence because we had only cross-sectional data. Therefore, longitudinal studies should be carried out to further clarify the problem. Third, future studies should combine electroencephalogram, magnetic resonance imaging and other research methods to explore the neural mechanism of inhibitory control in patients with different levels of dietary adherence to provide a theoretical basis for further intervention.

## Conclusions

In conclusion, this is the first study to present evidence of the relationship between inhibitory control and dietary adherence among patients with T2DM based on subjective and objective measures. We found that inhibitory control was a predictor of dietary adherence in patients with T2DM. Our study contributes to a deep understanding of the role of inhibitory control in dietary adherence, which provides a new perspective to further improve dietary adherence among patients with T2DM in clinical practice. Previous studies have reported the feasibility of nurse-led cognitive training to improve the self-management behavior of patients with T2DM. In the future, we can consider improving the dietary adherence of patients with T2DM based on inhibitory control training, which may be good news for patients with diabetes, especially for those with poor dietary adherence.

## Data Availability

The datasets generated and analyzed during the current study are available from the corresponding authors upon reasonable request.

## References

[1] Liu Y, Yu D, Luo J, Cai S, Ye P, Yao Z (2022). Self-reported dietary management behaviors and dietary intake among Chinese adults with diabetes: a population-based study. Nutrients.

[2] IDF Diabetes Atlas 2021. 2021. https://diabetesatlas.org/atlas/tenth-edition/. 35914061

[3] Zheng Y, Ley SH, Hu FB (2018). Global aetiology and epidemiology of type 2 diabetes mellitus and its complications. Nat Rev Endocrinol.

[4] Ajala O, English P, Pinkney J (2013). Systematic review and meta-analysis of different dietary approaches to the management of type 2 diabetes. Am J Clin Nutr.

[5] Raj GD, Hashemi Z, Soria Contreras DC, Babwik S, Maxwell D, Bell RC (2018). Adherence to diabetes dietary guidelines assessed using a validated questionnaire predicts glucose control in adults with type 2 diabetes. Can J Diabetes.

[6] American Diabetes Association. (2021). 6. Glycemic targets: Standards of medical care in diabetes—2021. Diabetes Care.

[7] Chinese Diabetes Society. (2021). Guidelines for prevention and treatment of type 2 diabetes in China (2020 ed). Int J Endocrinol Metab.

[8] Akbar H, Anderson D, Gallegos D (2015). Predicting intentions and behaviours in populations with or at-risk of diabetes: a systematic review. Prev Med Rep.

[9] Hawley JA, Sassone-Corsi P, Zierath JR (2020). Chrono-nutrition for the prevention and treatment of obesity and type 2 diabetes: from mice to men. Diabetologia.

[10] Egbert AH, Stockdale LA, Nicholson LM, Sroka A, Szpak V, Morrison RG (2022). Delicious and difficult to resist?: Inhibitory control differs in young women after exposure to food and non-food commercials. Appetite.

[11] Liu Y, Gao X, Zhao J, Zhang L, Chen H (2020). Neurocognitive correlates of food-related response inhibition in overweight/obese adults. Brain Topogr.

[12] Diamond A (2013). Executive functions. Annu Rev Psychol.

[13] Wu D, Zhou Y, Xu PB, Liu N, Sun KW, Xiao W (2022). Initial performance modulates the effects of cathodal transcranial direct current stimulation (tDCS) over the right dorsolateral prefrontal cortex on inhibitory control. Brain Res.

[14] Gelonch O, Garolera M, Valls J, Rosselló L, Pifarré J (2016). Executive function in fibromyalgia: comparing subjective and objective measures. Compr Psychiatry.

[15] Hamburger ER, Lyttle M, Compas BE, Jaser SS (2019). Performance-based and questionnaire measures of executive function in adolescents with type 1 diabetes. J Behav Med.

[16] Su YH. The brain mechanisms research of inhibition control between successful and failed restraint eaters. Chongqing: Southwest University; 2017.

[17] Guerrieri R, Nederkoorn C, Jansen A (2008). Interaction between impulsivity and a varied food environment: its influence on food intake and overweight. Int J Obes.

[18] Guerrieri R, Nederkoorn C, Jansen A (2007). How impulsiveness and variety influence food intake in a sample of healthy women. Appetite.

[19] Jasinska AJ, Yasuda M, Burant CF, Gregor N, Khatri S, Sweet M (2012). Impulsivity and inhibitory control deficits are associated with unhealthy eating in young adults. Appetite.

[20] Zhang XM, Luo Y, Liu Y, Yang C, Chen H (2019). Lack of conflict during food choice is associated with the failure of restrained eating. Eat Behav.

[21] Wyckoff EP, Evans BC, Manasse SM, Butryn ML, Forman EM (2017). Executive functioning and dietary intake: neurocognitive correlates of fruit, vegetable, and saturated fat intake in adults with obesity. Appetite.

[22] Adams RC, Button KS, Hickey L, Morrison S, Smith A, Bolus W (2021). Food-related inhibitory control training reduces food liking but not snacking frequency or weight in a large healthy adult sample. Appetite.

[23] Oomen D, Grol M, Spronk D, Booth C, Fox E (2018). Beating uncontrolled eating: training inhibitory control to reduce food intake and food cue sensitivity. Appetite.

[24] Ganor-Moscovitz N, Weinbach N, Canetti L, Kalanthroff E (2018). The effect of food-related stimuli on inhibition in high vs. low restrained eaters. Appetite.

[25] Manasse SM, Goldstein SP, Wyckoff E, Forman EM, Juarascio AS, Butryn ML (2016). Slowing down and taking a second look: Inhibitory deficits associated with binge eating are not food-specific. Appetite.

[26] Liu Z, Jiang J, Cai T, Zhang DL. A study of response inhibition in overweight/obesity people based on event-related potential. Front Psychol. 2022;13:826648. 10.3389/fpsyg.2022.826648 PMC8929195 35310211

[27] Hall PA, Fong GT. Temporal sself-regulation theory: integrating biological, psychological, and ecological determinants of health behavior performance. In: Hall P, editor. Social Neuroscience and Public Health. New York, NY: Springer New York; 2013, p. 35–53.

[28] Chinese Society of Endocrinology. (2021). Expert consensus on diabetic cognitive dysfunction. Chin J Diabetes Mellit.

[29] Wang LJ, Liu X, Lu CH, Wang HX, Liu Y, Lv N (2020). Application of AD8 combined and MOCA in detecting cognitive impairment in some middle-aged and elderly people undergoing physical examination. Med Innov.

[30] Zhao QL, Hou SN, Liang Y, Zhao J, Wang LM (2017). Dietary behavior compliance scale for patients with type 2 diabetes mellitus: development and validation. J Nurs Sci.

[31] Robert M, Peter K, Gerard A. BRIEF-A: behavior rating inventory of executive function-adult version: professional manual. Lutz, FL: Psychological Assessment Resources Inc.; 2005.

[32] Du Q, Qian Y, Wang Y (2010). Reliability and validity of the behavior rating inventory of executive function-adult version self-report form in China. J Chin Ment Health.

[33] Raud L, Westerhausen R, Dooley N, Huster RJ (2020). Differences in unity: The go/no-go and stop signal tasks rely on different mechanisms. Neuroimage.

[34] Fang J, Zhu Y, Zhao W, Zhang B, Wang X (2013). Research progress of stop signal task in mental illness. Chin J Nerv Ment Dis.

[35] Davey S, Halberstadt J, Bell E (2022). Where is an emotion? Greater interference in a gut-focused visceroception group undertaking an emotional stop-signal task. N Ideas Psychol.

[36] Verbruggen F, Aron AR, Band GP, Beste C, Bissett PG, Brockett AT (2019). A consensus guide to capturing the ability to inhibit actions and impulsive behaviors in the stop-signal task. Elife.

[37] Luo X, Gu J, Zheng Y, Zhou X (2022). Making a saccade enhances Stroop and Simon conflict control. Attention Percept Psychophys.

[38] Sabaté E, Sabaté E, editors. Adherence to long-term therapies: evidence for action. Geneva: World Health Organization; 2003. 14562485

[39] Mahone EM, Martin R, Kates WR, Hay T, Horská A (2009). Neuroimaging correlates of parent ratings of working memory in typically developing children. J Int Neuropsychol Soc.

[40] Hagen E, Erga AH, Hagen KP, Nesvåg SM, McKay JR, Lundervold AJ (2016). Assessment of executive function in patients with substance use disorder: a comparison of inventory- and performance-based assessment. J Subst Abus Treat.

[41] Zhu GY, Liu AN, Ding CL, Sun XH, Zheng HY, Chen GH (2020). Characteristics of inhibitory control in type 2 diabetic patients with different levels of dietary adherence. Chin Gen Pract.

[42] Batterink L, Yokum S, Stice E (2010). Body mass correlates inversely with inhibitory control in response to food among adolescent girls: an fMRI study. Neuroimage.

[43] Hall PA, Lowe C, Vincent C (2014). Executive control resources and snack food consumption in the presence of restraining versus facilitating cues. J Behav Med.

[44] Zhang X, Chen S, Chen H, Gu Y, Xu W (2017). General and food-specific inhibitory control as moderators of the effects of the impulsive systems on food choices. Front Psychol.

